# Economic downturns and male cesarean deliveries: a time-series test of the economic stress hypothesis

**DOI:** 10.1186/1471-2393-14-198

**Published:** 2014-06-07

**Authors:** Tim A Bruckner, Yvonne W Cheng, Amrita Singh, Aaron B Caughey

**Affiliations:** 1 Public Health & Planning, Policy and Design, University of California, Irvine, 202 Social Ecology I, Irvine, CA 92697-7075, USA; 2 Center for Clinical and Policy Perinatal Research, University of California, San Francisco, 500 Parnassus Ave, San Francisco, California 94143, USA; 3 Department of Obstetrics, Gynecology, and Reproductive Sciences, University of California, San Francisco, 500 Parnassus Ave, San Francisco, California 94143, USA; 4 Department of Obstetrics and Gynecology, Oregon Health & Science University, 3181 S.W. Sam Jackson Park Rd, Portland, Oregon, USA; 5 Center for Women’s Health, Oregon Health & Science University, 3181 S.W. Sam Jackson Park Rd, Portland, Oregon 97239-3098, USA

**Keywords:** Cesarean delivery, Economic stress, Fetal distress, Male sensitivity

## Abstract

**Background:**

In light of the recent Great Recession, increasing attention has focused on the health consequences of economic downturns. The perinatal literature does not converge on whether ambient economic declines threaten the health of cohorts in gestation. We set out to test the economic stress hypothesis that the monthly count of cesarean deliveries (CD), which may gauge the level of fetal distress in a population, rises after the economy declines. We focus on male CD since the literature reports that male more than female fetuses appear sensitive to stressors *in utero*.

**Methods:**

We tested our ecological hypothesis in California for 228 months from January 1989 to December 2007, the most recent data available to us at the time of our tests. We used as the independent variable the Bureau of Labor Statistics unadjusted total state employment series. Time-series methods controlled for patterns of male CD over time. We also adjusted for the monthly count of female CD, which controls for well-characterized factors (e.g., medical-legal environment, changing risk profile of births) that affect CD but are shared across infant sex.

**Results:**

Findings support the economic stress hypothesis in that male CD increases above its expected value one month after employment declines (employment coefficient = -24.09, standard error = 11.88, p = .04). Additional exploratory analyses at the metropolitan level indicate that findings in Los Angeles and Orange Counties appear to drive the State-level relation.

**Conclusions:**

Contracting economies may perturb the health of male more than female fetuses sufficiently enough to warrant more CD. Male relative to female CD may sensitively gauge the cohort health of gestations.

## Background

The recent “Great Recession” serves as a reminder that the economy acts as a dynamic stressor that affects us all. The largest sustained rise in unemployment in the United States in over a quarter of a century [1], coupled with a fall in the value of homes and investments, has renewed interest in knowing whether the incidence of illness increases when the economy declines [2,3].

Most of the literature that reports a connection between economic decline and illness, although not without detractors [4], invokes the well-established “economy as a stressor” hypothesis [5-9]. This hypothesis asserts that adaptations to unwanted changes in the ambient environment may disrupt normal behavior and physiology which could, in turn, induce health sequelae. Examples of responses to economic downturns include undesirable economic (e.g., job loss, difficulty paying bills) or non-economic (e.g., family problems, change of residence) life events, all of which may prove stressful and increase the incidence of illness [6,10-12]. Additionally, even among those who remain working, anxiety, depression and poor self-reported health increase following economic downturns [13,14]. This stress response among workers is noteworthy because persons continuously employed during economic contraction far outnumber those who lose jobs.

Reports in California, Sweden, and Germany find that the secondary sex ratio (i.e., the odds of a live male birth) falls following declines in employment [15-17]. One explanation for this association includes that economic decline may increase corticosteroid production among pregnant mothers and affect fetal development. Male fetuses greater than 20 weeks of gestation reportedly react more sensitively than do female fetuses to these maternal corticosteroids [18,19]. This heightened sensitivity in the late 2^nd^ and 3^rd^ trimester may, following ambient stressors, jeopardize the viability of male more than female fetuses. Recent analyses support this heightened male sensitivity in that male relative to female fetal loss increases following economic downturns [20] and the terrorist attacks of September 11, 2001 [21]. A test using births in Sweden further indicates that male infants in particular show a greater incidence of very low birth weight after economic decline [22].

The relevance of the sex ratio and male birth weight results to maternal and child health would gain support if the economic stressor that reduces male fetal viability also increases the risk of health sequelae. We believe that the incidence of cesarean delivery (CD), the most commonly performed inpatient surgery in women in the United States (~1.2 million procedures per year) [23] represents one important clinical outcome [24-26] that may respond to economic downturns. One indication for CD, for example, involves nonreassuring fetal heart rate tracing, or “fetal distress” [27]. If the same physiological process which precedes greater male fetal loss also increases the risk of fetal compromise, then the rate of CD for male fetal distress may increase, thereby elevating CD for males overall [28].

We test the hypothesis that the risk of CD will increase when the economy worsens. We test this hypothesis in California, the most populous of the United States. Whereas California holds a large and diverse economic base, the Legislature enacts policies and regulations at the State level that distinctly affect the State’s economic situation. Previous literature, moreover, finds that the California birth cohort responds to the State’s economic downturns [20].

Consistent with the literature which reports heightened male fetal sensitivity to economic downturns, we focus our test on male births [17,20,22]. Importantly, we adjust for factors that affect male and female CD equally (e.g., clinical culture, “delivery on demand”) by including the monthly count of female CD as a control variable. Our analysis contributes to the literature in two ways. First, evidence in favor of our hypothesis would support that males more than females respond sensitively to ambient stressors *in utero.* Second, findings could inform the debate regarding whether, and to what extent, indicators of population health respond to macroeconomic change.

## Methods

### Variables and data

The California Department of Health Services maintains a birth file for all infants born in California or to California residents during a given calendar year. We acquired these data for the 228 months spanning from January 1989 to December 2007. This time frame represents the longest data series with consistent collection methodology available to us at the time of our test. The California Department of Health Services codes the data according to uniform specifications, performs rigorous statistical quality checks, carefully reviews and edits the birth file [29]. The reporting of births in California is nearly 100 percent complete [29]. We used de-identified, publicly available, aggregate level vital statistics data. Therefore, the study qualified as exempt from Human Subjects Review, as outlined by the regulations of the State of California Department of Public Health.

We classified a cesarean delivery (CD) as a live birth delivered by either a primary or repeat cesarean section. We excluded stillbirths from the analysis. We also excluded infants with missing or unknown data on sex or mode of delivery.

We retrieved the Bureau of Labor Statistics unadjusted total employment series to gauge the status of the economy in California [30]. Using these data facilitates replication of our analysis in other states because the Bureau makes these employment data freely available. We chose the total employment series because a decline in employed persons indicates that a fraction of the population has lost wages, tips, or other income and that this loss may ripple through the population. We use this measure to avoid ambiguity associated with other economic measures (e.g., unemployment rate) devised by labor economists. The unemployment rate, for example, measures the number of persons who report wanting to work but have no job. The unemployment rate, however, remains subject to ambiguous interpretation since it often captures beneficial employment trends. For example, the unemployment rate may increase with total employment because economic expansions often compel more persons to enter the labor market or leave their current jobs to pursue more favorable employment opportunities [31]. This circumstance would not cause undesirable economic and non-economic events that, as described in the Introduction, could perturb the physiology of pregnant women.

### Analysis

**Figure 1:**
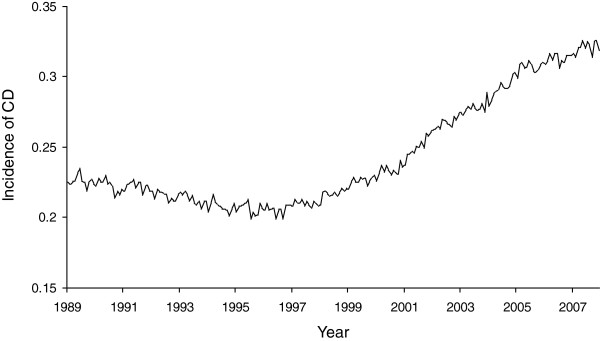
Incidence of Cesarean Delivery (per live births) in California for 228 months beginning January, 1989.

We used traditional autoregressive integrated moving average (ARIMA) time-series methods to test the ecological proposition that the monthly count of CD among male births rises above expected values when monthly state employment falls [32,33].The monthly count of CD in California exhibit seasonality and other temporal patterns, such as a strong upward trend after 1997. These patterns, known as autocorrelation, violate the assumption of correlational tests because the expected value of CD is not the mean from past months. As indicated by Figure 1, we would not expect the count of CD in 2007 to equal the mean count of CD from 1989 to 2006. This secular trend, and other patterns, may induce a spurious relation between economic decline and CD if the patterns in CD were not statistically controlled.

Time-series methods devised by Box and Jenkins [32] address this autocorrelation problem. Researchers have devised routines that empirically identify and remove patterns in the outcome variable. This data-driven approach identifies and removes autocorrelation such that (1) the expected value of the residuals of the outcome variable is zero and (2) the monthly observations are statistically independent of one another. In our test, removing autocorrelation from CD in male births before testing the relation of employment decline also minimizes the risk of confounding that could arise from a shared pattern between employment and high or low values of CD in males.

We used the strategies devised by Dickey and Fuller [33] as well as Box and Jenkins [32] to identify and model temporal patterns in the monthly male CD in California for the 228 months beginning January 1989. The Dickey-Fuller test detects the trends and seasonality, whereas Box and Jenkins routines model these patterns and the tendency of a series to remain elevated or depressed, or to oscillate, after high or low values.

We then built on the above strategy with a more rigorous approach from the perinatal epidemiology literature [34]. We included the monthly count of CD in female births as an independent variable in the time-series equation. Including the count of female CD as a control variable removes confounding by forces that affect the incidence of CD equally across sex (e.g., maternal age, ethnicity, changes in clinical culture, delivery on demand).

We proceeded through five steps. First, we estimated initial models, which include the control variable of the count of female CD, using software from Scientific Computing Associates (version 5.4.6, SCA Corp., Villa Park, IL). Second, we inspected the error term for autocorrelation and, if needed, inserted Box-Jenkins ARIMA parameters to remove any remaining temporal patterns in male CD. Third, we added the independent variable (i.e., monthly change in employment lagged at 0, 1 and 2 months) to the best-fitting Box-Jenkins models of male CD. We specified employment with no lag (i.e., employment the same month as CD) as well as in lags of 1 and 2 months (i.e., employment one and two months before CD) to ensure capturing any delayed associations induced near the end stage of gestation. This time lag is consistent with literature reporting male-specific responses to population shocks within a few months [17,20,21]. Fourth, we inspected the residual values of the error term to ensure that they exhibited no temporal patterns. Fifth, we performed sensitivity tests and assessed the stability of results to outliers and alternative assumptions.

## Results

**Figure 2:**
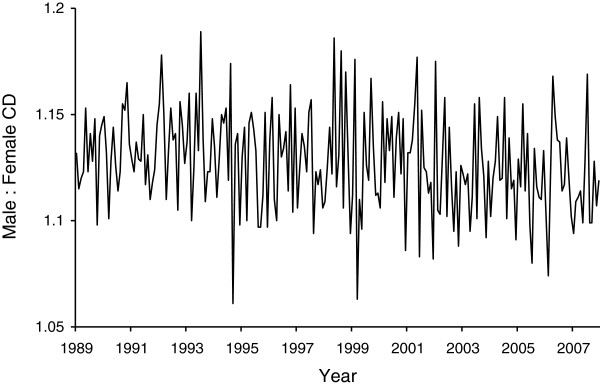
Ratio of Male to Female Cesarean Deliveries in California for 228 months beginning January, 1989.

**Figure 3:**
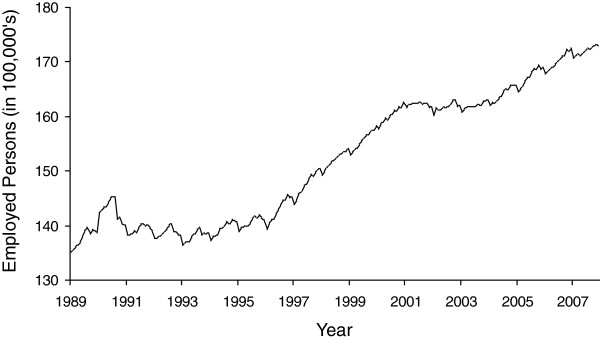
Employed persons (in 100,000 s) in California for 228 months beginning January, 1989.

**Figure 4:**
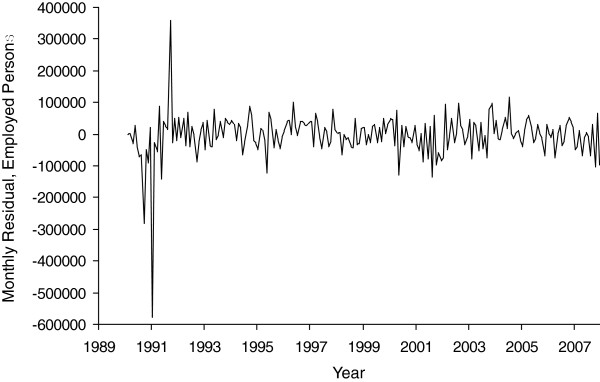
**Monthly change in employed persons after removal of trend and seasonal non-stationarity in California for 228 months beginning January, 1989.** (The first 13 months are lost due to time-series modeling).

Table 1 provides descriptive characteristics of the 10,555,024 live births in California over the study period. Hospitals classified almost a quarter of all births (24.4 percent) as a cesarean delivery. Infants born to Hispanic mothers accounted for 47 percent of all births. Nine percent of live births were delivered preterm.Figure 1 displays the monthly incidence of CD over the test period, which indicates a slight decline until 1997 and a steady rise thereafter. Figure 2 shows the ratio of male to female CD. Unlike the overall incidence of CD, the ratio of male to female CD variable does not exhibit an upward trend. The fact that the mean (1.127) is greater than the birth sex ratio (1.048), however, indicates that male infants appear at greater risk than female infants to undergo CD.Figure 3 shows total employment in California for the same months. As expected, monthly values move around a general upward trend which itself exhibits variability and breaks. The Dickey-Fuller test indicated non-stationarity which required taking the first difference (i.e., subtracting employment at month t + 1 from that at month t). Employment also showed such strong seasonality to warrant differencing at lag 12 (i.e., subtracting employment at month t + 12 from that at month t). After this differencing, a negative value of the employment variable indicates “economic decline” since it captures an unexpected fall in employment, controlling for seasonality and trend, relative to employment in the previous month. We plot this differenced series in Figure 4, which represents the independent variable in our test. This series shows neither trend nor seasonality, includes months of severe economic decline (e.g., maximum loss = -576,000 jobs in January 1991), and is statistically independent of California’s estimated population size.

**Table 1:** Descriptive characteristics of live births in California, 1989–2007

	**N**	**%**
Cesarean Delivery	2,575,801	24.40
Singleton	10,280,994	97.40
Male infant	5,400,155	51.16
Preterm (<37 completed weeks)	958,280	9.08
Maternal Age		
< 18 years	424,614	4.02
18-25 years	3,820,752	36.20
26-34 years	4,781,611	45.31
≥ 35 years	1,526,339	14.46
Maternal Race/Ethnicity		
Non-hispanic white	3,616,819	34.27
Non-hispanic black	705,735	6.69
Hispanic	4,958,368	46.98
Asian/Pacific Islander	1,147,139	10.87
Other	126,923	1.20

Note: column totals may not sum to 100% due to rounding.

Table 2 displays results from the final time-series equation (sample size is 228 months). The count of female CD strongly predicts the count of male CD in the same month. Box-Jenkins modeling indicated no temporal patterning in male CD after we included female CD as a control variable. As a result, the equation required no time-series error term.

**Table 2:** Time-series results estimating the monthly count of male Cesarean Deliveries (CD) in California as a function of monthly female CD, monthly State employment, and autocorrelation (sample size = 228 months)

	**Monthly Count of Male CD**
Constant	179.18 (47.26)*
Count of Female CD	1.093 (.009)***
Employment (in 100,000 s) at:	
Same Month of CD	-4.57 (12.14)
1 Month before CD	-24.09 (11.88)*
2 Months before CD	-6.00 (12.21)
Differencing	None
Autoregressive Parameters	None
Moving Average Parameters	None

*p < .05; *p < .01; ***p < .001; 2- sided test.

Standard errors appear in parentheses.

Findings support the economic stress hypothesis in that employment moves inversely with the ratio of male to female CD. We observe an inverse association at lag 1 month (coef. = -24.09, standard error [SE] = 11.88, p = .043), which implies that a decline in 100,000 employed persons precedes by one month an increase in 24.09 male (relative to female) CD.

We performed several quality checks to assess the robustness of our results. First, we inspected the error term of the final equation for autocorrelation. None was found. Second, we performed outlier detection and correction routines recommended by Chang and colleagues [35]. These routines control for the possibility that unusually high or low values of the dependent variable drive the findings. We detected four outliers; controlling them did not change inference for the employment coefficients, and the negative association at lag 1 month remained similar to that of the original test (coef. = -22.79, SE = 10.84, p = .037). Third, we examined the possibility that non-constant variance of male relative to female CD produced a type I error. We homogenized variance in the series by transforming values to their natural log and re-estimated the test equation. Results remained essentially unchanged. Finally, we examined whether use of the count of male CD, rather than the popular log-odds (i.e., logit) transformation, may have affected the results. Conversion of the outcome variable to the logit of male CD did not change inference from the original results shown in Table 2.

To help the reader gauge the size of the discovered findings, we calculated the number of CD among males statistically attributable to declines in employment. Over the 228 months, employment fell below expected levels in 71 months. The mean loss during these months was 67,625 jobs. Applying this value to the lag 1 month coefficient in Table 2 implies an excess of 16 male CDs per month (range: 1 to 96 additional male CD per month) in which employment fell. In sum, this equates to 1,156 more CD in male births than expected across all months of employment decline.

### Exploration

California’s large population and diverse economy implies that our State-level findings represent an average effect across its distinct metropolitan regions. To explore whether findings differ across the State, we assessed the economy/male CD relation in the two most populous metropolitan statistical areas (MSAs) in California: Los Angeles and San Francisco. We chose MSAs as the geographic unit of analysis given that the U.S. Office of Management and Budget designates residents in an MSA as sharing social and economic integration with the metropolitan center (https://www.census.gov/population/metro/). The Los Angeles MSA includes Los Angeles and Orange Counties and accounts for 40 percent of all births in California. The San Francisco MSA includes Alameda, Contra Costa, Marin, San Francisco, and San Mateo counties and accounts for 9.8 percent of all births in California. We proceeded through the steps, described in the Methods, to remove autocorrelation in the dependent variable series and then test the relation between employment change and male CD. The Los Angeles findings appear similar to the California test, except that the negative lag 1 month coefficient for employment is stronger (coef. = -52.07, SE = 18.40, p = .005). We, however, cannot reject the null in the San Francisco test, although the lag 1 month coefficient is negative (coef. = -21.64, SE = 25.45, p = .40). This exploration indicates that the relatively strong Los Angeles MSA result appears to drive the State-level findings that male CD rises after the economy declines.

## Discussion

Time-series analysis of all births in California from 1989 to 2007 indicates that male CD rises above expected levels one month after employment falls. Results support the economic stress hypothesis in that male more than female fetuses may react sensitively to ambient stressors *in utero*. This possible increased male sensitivity to economic downturns may induce clinically important sequelae such as CD.

Findings appear consistent with reports of perturbed sex ratios and very low birth weight among males following economic contraction [17,20,22]. Declines in employment may increase the production of corticosteroids among gravid females which, in turn, could adversely affect male more than female fetuses. Although the maternal response to ambient drops in employment may not appear deleterious enough to induce fetal loss, the response may elicit clinical signs of distress upon which medical staff may intervene.

Statistical control for the monthly count of female CD adjusts for unmeasured confounders that affect the incidence of CD equally in both sexes (e.g., maternal age, race/ethnicity, clinical culture). For this reason, any factor that influences the risk of male and female CD equally cannot confound our results. Findings also cannot arise from trend, seasonality, or other patterns in male CD because we detected no such autocorrelation after adjustment of female CD. We apply a conservative time-series method in that only statistically unexpected values in the dependent variable remain available for explanation by employment change. Consequently, our estimates of excess male CDs statistically attributable to employment declines may approximate the lower bound of the true effect. We, nevertheless, acknowledge that the small magnitude of the male CD result holds implications more for the theory of male fetal frailty than for clinical interventions.

The discovered 1-month delayed effect appears consistent with prior literature regarding the timing of acute stressors and birth outcomes [20,22]. In addition, the 1-month lag avoids measurement error inherent in the 0-month lag coefficient in which births early in the month may occur prior to economic downturns later in that month. The physiology of pregnant women, moreover, may require a sustained stress response to economic downturns that manifests in the adverse delivery outcome only in the subsequent month.

One mechanism that may perturb the trajectory of male gestations during economic downturns involves reduced maternal sleep. Sleep disruptions, especially among women, occur more frequently among persons with anxiety and depressive symptoms― two conditions which appear elevated during economic decline [36,37]. Researchers further find that poor sleep in the late third trimester varies positively with of the incidence of cesarean delivery [38]. We await future research on the relation between sleep and perinatal outcomes.

Limitations of our approach include that we cannot know if results generalize outside of California. Over our test period, we could not acquire data with appropriate identifiers from other states. In addition, undercount of birth data in California by sources other than the California Department of Vital Statistics (http://wonder.cdc.gov/natality-current.html) precluded a time-series test of more recent years including the Great Recession. We anticipate receipt of a data file with internally consistent collection methods in the future. Despite these limitations, we note that the sheer volume of births analyzed (10 million over the test period) exceeds that of many developed countries. Our exploration of the Los Angeles and San Francisco MSAs also shows regional differences in the economy/male CD relation. The reader, therefore, should not use our California results to make inference to specific regions. We encourage more careful examination of local variation in clinical culture and economic circumstances to help explain the Los Angeles and San Francisco results.

We also caution against using the employment coefficient to estimate the impact of individual employment loss on male CD. A change in employed persons in California gauges ambient economic circumstances which researchers should consider as similar to studies of the effect of climate change on health. Individual-level studies on pregnant women before and after economic downturns would complement our ecological test and may illuminate behavioral and biological mechanisms by which mothers respond to unwanted economic changes.

We also note that CD represents one of the many indicators of fetal health that may respond to economic change [39-42]. Margerison-Zilko provides an excellent review of the literature concerned with the economy and birth outcomes [42]. The review highlights several indicators used in studies of economic antecedents of fetal health (e.g., birth weight, small-for-gestational age, preterm, fetal loss). Whereas much of this work suggests that birth weight falls after the economy contracts, the field does not reach a consensus. One analysis shows the counterintuitive result of improved birth weight when the economy declines [40], and research on adults more generally also reports lower mortality during recessions [4]. We concur with Margerison-Zilko [42] on the need for improved study designs, as well as replication efforts, in this emerging perinatal epidemiology field to bolster its internal and external validity.

Of the literature reviewed above, scant research focuses on male fetal viability. Absent data on fetal distress in the birth file, we used male CD as a clinically relevant proxy for male fetal distress. We acknowledge that other candidate biomarkers for fetal viability have been proposed. Researchers, for instance, report that maternal hCG during pregnancy, especially among male gestations, responds to fluctuations in the ambient economy [43]. We encourage further investigation― especially among males― of whether the current economic recession disrupts the trajectory of cohorts in gestation.

## Conclusions

Much literature finds that clinical culture, the medical-legal environment, and patient preferences drive the temporal variation in CD [44-47]. We, however, know of no research other than our report that examines whether ambient stressors affect CD. Our findings suggest that clinicians may respond to the risk profile of cohorts which varies over time. The observation that clinicians adapt practice to the time-varying needs of birth cohorts subjected to stressors *in utero* should reassure health professionals that increasingly expect evidence-based care. We await future replication efforts to determine the generalizability of the California results to other places and times (see, for example, http://wonder.cdc.gov/natality-current.html).

California recently faced a $54 billion budget deficit and the longest extended drop in employment since 1975 [48]. This climate renewed interest in quantifying the health implications of economic change. We report that one unanticipated consequence of economic downturns involves an increased risk of male CD. Although the magnitude of the discovered effect does not appear sufficiently large to warrant closer clinical scrutiny of male gestations at high risk of fetal distress, results further support increased male reactivity to stressors *in utero.* Findings also indicate that clinical practice may respond to this elevated reactivity. Our results should encourage further investigation on the perinatal sequelae of economic downturns.

## Abbreviations

CD: Cesarean delivery; ARIMA: Autoregressive integrated moving average; SE: Standard error.

## Competing interests

The authors declare that they have no competing interests.

## Authors’ contributions

TAB co-designed the research question, retrieved the data, performed the statistical analysis, and served as the lead author of the manuscript. YWC assisted with the data analysis and interpretation of results, wrote sections of the Background, and edited all sections. AS assisted with writing the Background and edited all sections. ABC co-designed the research question, interpreted the results, and wrote sections of the Discussion. All authors read and approved the final manuscript.

## Pre-publication history

The pre-publication history for this paper can be accessed here:

http://www.biomedcentral.com/1471-2393/14/198/prepub
